# Characterization of a novel B-cell epitope in the structural protein of goose astrovirus 1 and its application in serological detection

**DOI:** 10.1016/j.psj.2025.105674

**Published:** 2025-08-12

**Authors:** Anping Wang, Li Liu, Qingkang Zhou, Xiaolu Zhang, Zhi Wu, Shanyuan Zhu

**Affiliations:** Jiangsu Agri-Animal Husbandry Vocational College, Jiangsu Key Laboratory for High-Tech Research and Development of Veterinary Biopharmaceuticals, Taizhou 225300, PR China

**Keywords:** Goose astrovirus 1, Monoclonal antibody, Epitope, Indirect competitive ELISA

## Abstract

Goose astrovirus 1 (**GAstV-1**) is an emerging pathogen responsible for gosling gout and has caused substantial economic losses in the goose industry, particularly during frequent co-infections with goose astrovirus 2 (**GAstV-2**) that exacerbate pathogenicity. Despite advances in GAstV-2 research, the lack of GAstV-1-specific monoclonal antibodies (**mAbs**) and defined epitopes has hindered the development of targeted diagnostic tools and mechanistic studies. To address this gap, we generated the first GAstV-1-specific mAb (A5A1) by immunizing mice with prokaryotically expressed recombinant ORF2 protein. Western blotting (**WB**), immunofluorescence assay (**IFA**), and immunohistochemistry (**IHC**) confirmed A5A1’s strict specificity for GAstV-1. Crucially, we identified a linear B-cell epitope (^394^SNREVQITQL^403^) within the conserved P1 domain of ORF2. Structural modeling revealed its surface-exposed β-sheet conformation, while sequence alignment demonstrated high conservation among GAstV-1 strains but marked divergence from GAstV-2 and other avian astrovirus (**AAstV**) strains, highlighting its diagnostic utility. Leveraging A5A1, we developed an indirect competitive ELISA (**icELISA**) for GAstV-1 antibody detection. The assay demonstrated high sensitivity (detection limit: 1:128 serum dilution), robust reproducibility (intra-/inter-assay CVs <10 %), and exceptional specificity (no cross-reactivity with GAstV-2 or other avian pathogens). Clinical validation using 80 field sera showed 87.5 % concordance with IFA, confirming its reliability. Collectively, this study delivers the first GAstV-1-specific mAb, a novel diagnostic epitope, and a robust serological tool, thereby advancing GAstV-1 pathogenesis research and enabling effective disease surveillance.

## Introduction

Goose astrovirus (**GAstV**), a member of the genus *Avastrovirus* within the family *Astroviridae*, is a single-stranded RNA virus with non-enveloped icosahedral symmetry. Phylogenetic analyses classify GAstVs into two genetically distinct genotypes: goose astrovirus 1 (**GAstV-1**) and goose astrovirus 2 (**GAstV-2**), which exhibit approximately 60 % genomic nucleotide homology (Fei et al., 2022; Zhu and Sun, 2022). The GAstV-1 genome contains three open reading frames (**ORFs**), with ORF2 encoding the synthesis of the structural protein essential for viral assembly and immunogenicity. The ORF2 structural protein undergoes proteolytic maturation: in human astroviruses (**HAstVs**), the ∼90 kDa ORF2 protein precursor (**VP90**) is intracellularly cleaved by caspases, removing the C-terminal acidic domain to yield a ∼70 kDa product (**VP70**). Following virion release, VP70 is further processed extracellularly by various proteases into two major protein fragments: VP34 and VP27. VP34, containing the conserved S and P1 domains, forms the rigid capsid core, whereas VP27, carrying the hypervariable P2 domain, constitutes surface spike on the virion (Arias and DuBois, 2017).

As an emerging avian pathogen, GAstV-1 substantially threatens waterfowl health and global goose production systems. The virus primarily targets the gastrointestinal tract, leading to symptoms such as diarrhea, growth retardation, and high mortality in young goslings (Wang et al., 2025). Although epidemiological surveys indicate that GAstV-2 is the predominant circulating strain associated with gosling gout, recent years have witnessed a rise in GAstV-1 infections (Fu et al., 2022; Wang et al., 2022a). Moreover, the increasing prevalence of co-infections with GAstV-2 has been shown to exacerbate gout symptoms and elevate mortality rates in goslings (Chen et al., 2024; Zhou et al., 2024). Current control strategies primarily rely on biosecurity measures, as no commercial vaccines are available. This underscores the critical need for robust diagnostic tools.

To date, the development of diagnostic methods for GAstV has predominantly focused on GAstV-2 (Wang et al., 2022b; He et al., 2023; Zhang et al., 2023), with relatively limited reports on diagnostic approaches for GAstV-1. Existing nucleic acid-based methods, such as reverse-transcription (**RT**)-PCR and reverse-transcription loop-mediated isothermal amplification (**RT-LAMP**), are insufficient for assessing herd immunity or vaccine efficacy (Yuan et al., 2018; Yu et al., 2020; Wang et al., 2022c). Using monoclonal antibodies (**mAbs**) targeting structural proteins represents a promising strategy to address these gaps. However, no GAstV-1-specific mAbs or epitopes have been reported thus far. In this study, we describe the generation of a GAstV-1 ORF2-specific mAb, the identification of its linear B-cell epitope, and the development of a novel indirect competitive ELISA (**icELISA**) for the detection of GAstV-1 antibodies. Our findings fill critical knowledge gaps regarding GAstV-1 antigenicity and provide essential tools for epidemiological monitoring and vaccine design.

## Materials and methods

### Ethics statement

Experimental protocols strictly complied with the Chinese National Guidelines for Laboratory Animal Welfare and were formally authorized by the Institutional Animal Care Committee at Jiangsu Agri-animal Husbandry Vocational College (Approval ID: JSAHVC-2024-35).

### Virus and cells

The GAstV-1 TZ03 strain (GenBank accession No. MW353015) was stored in our laboratory and propagated in 9-day-old goose embryos. Virus handling complied with BSL-2 containment protocols (WHO guidelines), utilizing Class II biosafety cabinets, autoclaving of liquid waste, and appropriate personal protective equipment. Primary goose embryonic kidney (**GEK**) cells were isolated from 18-day-old goose embryos using established protocols (Guo et al., 2024), and maintained in Dulbecco's Modified Eagle Medium (**DMEM**; Gibco, USA) supplemented with 10 % fetal bovine serum (**FBS**; Lonsera, China), 100 U/mL penicillin, and 10 μg/mL streptomycin at 37°C under 5 % CO₂. The human embryonic kidney 293A cell line was cultured in the same conditions.

### Expression and purification of recombinant GAstV-1 ORF2 protein

To obtain the recombinant GAstV-1 ORF2 protein, the ORF2 whole gene of the TZ03 strain was amplified using a pair of specific primers: ORF2-F (5’- TAC-GGATCCATGGCCGACAAGGTCACTGTC-3’) and ORF2-R (5’- GCACTCGAG-TTAATCAAACTCTTGTCCGCC-3’). The amplified products were cloned into the pET-30a vector, and subsequently transformed into *E. coli* BL21(DE3) cells (Vazyme, Nanjing, China). Expression of the recombinant protein was induced by adding 0.2 mM isopropyl β-D-1-thiogalactopyranoside (**IPTG**) and incubating the cells at 20°C for 18 h. Following expression, the recombinant ORF2 protein was purified under denaturing conditions (8 M urea, 20 mM imidazole) using His-tag affinity chromatography with a HisGrip Master column (Vazyme). The purity of the recombinant protein was assessed by SDS-PAGE, Western blotting (**WB**) analysis with anti-His tag antibodies (Cwbio, Taizhou, China) confirmed the identity of the purified protein. The purified recombinant ORF2 protein concentration was determined using a BCA protein assay kit (Beyotime, Shanghai, China).

### Generation and characterization of monoclonal antibody (mAb)

Female BALB/c mice (6 weeks old; Huachuang Sino, Taizhou, China) were immunized subcutaneously with 50 μg purified ORF2 protein emulsified in Freund’s complete adjuvant (Sigma-Aldrich, Missoula, USA), followed by three boosts with Freund’s incomplete adjuvant at 14-day intervals. Serum antibody titers were monitored by indirect ELISA (**iELISA**). Splenocytes were harvested from mice with high serum titers and fused with SP2/0 myeloma cells using 50 % polyethylene glycol (**PEG** 1500; Roche, Basel, Switzerland). Hybridomas were selected and cultured in HAT medium followed by HT medium, both supplemented with 15 % FBS. Supernatants were screened by iELISA using ORF2-coated plates (0.5 μg/mL in PBS). Positive hybridomas underwent three rounds of subcloning by limiting dilution to establish monoclonal cell lines. The isotype of mAbs was determined using a commercial ELISA kit (Mlbio, Shanghai, China). For large-scale production, selected hybridomas were injected intraperitoneally into BALB/c mice. Ascitic fluid was harvested and mAbs were purified using HiTrap Protein G HP columns (Merck, USA) according to manufacturer protocols.

### Serological assays

For WB, samples were analyzed by SDS-PAGE using a 10 % gel and subsequently transferred onto a polyvinylidene fluoride membrane. The membrane was blocked overnight with 100 μl of 5 % skim milk in PBS (blocking buffer) at 4°C. After blocking, the membrane was incubated with either a His-tag antibody (Cwbio) or a mAb diluted in blocking buffer at 37°C for 2 h. The bound antibodies were detected using horseradish peroxidase (**HRP**)-conjugated goat anti-mouse IgG (KPL, Gaithersburg, MD, USA) diluted 1:5000 in blocking buffer. The signal was visualized using standard enhanced chemiluminescence (**ECL**) detection. For immunofluorescence assay (**IFA**), approximately 2 × 10⁵ GEK cells were plated in a 24-well plate and infected with the GAstV-1 TZ03 strain. Three days post-infection, cellular samples underwent fixation in 4 % paraformaldehyde solution and subsequent permeabilization treatment using 0.5 % Triton X-100. IFA was performed through sequential incubation with a primary mAb (1:2000) and fluorescein isothiocyanate (**FITC**)-labeled goat anti-mouse IgG secondary antibodies (1:5,000; KPL). Fluorescence images were captured using an inverted fluorescence microscope. For immunohistochemical (**IHC**) analysis, one-day-old healthy goslings (supplied by Jinpeng Co., Jiangsu Province, China) were orally inoculated with 0.3 mL of GAstV-1 TZ03 strain (0.3 × 10^3.25^ TCID50/goose), with PBS-inoculated goslings serving as negative controls. Five days post-inoculation, the goslings were euthanized using CO₂. Immediately post-euthanasia, target organs (small intestine, lungs, and heart) were dissected and immersion-fixed in 10 % neutral buffered formalin (10:1 v/v fixative: tissue) at 4°C for 48 h. Tissues were dehydrated through graded ethanol concentrations (70 %-100 %), cleared in xylene, and embedded in paraffin at 60°C. Sections (4-5 μm) were dried overnight at 37°C and stored with desiccant at 4°C. For IHC staining, slides were deparaffinized in xylene, rehydrated through descending ethanol concentrations, subjected to heat-mediated antigen retrieval using 10 mM citrate buffer (pH 6.0) at 95-100°C for 20 min, quenched with 3 % H₂O₂ in methanol for 10 min, and blocked with 5 % normal serum. Immunostaining was performed according to the previous method (Wang et al., 2025), using mAb A5A1 (1:500) as the primary antibody.

### B cell epitope mapping

Three fragments (S, P1, and P2 domains) of the GAstV-1 TZ03 ORF2 gene and a truncated C-terminal region of the P1 domain were amplified using the specific primers (Table 1). The amplified gene fragments were cloned into the eukaryotic expression vector pSC-FLAG using the ClonExpress™ II kit (Vazyme), resulting in a series of recombinants with ORF2 gene fragments of various lengths. Approximately 2 × 10⁵ 293A cells were seeded in a 24-well plate and transfected with the recombinant plasmids. Two days post-transfection, IFA was performed as described above, using the mAbs to be tested or the anti-FLAG mAb (1:2000; Sigma) as the primary antibodies.

**Table 1:** Primers used for amplification of goose astrovirus 1 ORF2 truncations.

Primer name	Direction	Sequence (5ʹ-3ʹ)
S-263-F	Forward	GACAAAGGCCGGCCAGAATTCGCCGACAAGGTCACTGTCT
S-263-R	Reverse	GGCTCGAGAGGCCTTGAATTCTTATTTTGGTGTATAGTTGGAAAAC
P2-294-F	Forward	GACAAAGGCCGGCCAGAATTCCAGAACCTACCACTCATCT
P2-294-R	Reverse	GGCTCGAGAGGCCTTGAATTCTTATGAAGTTTTGAGGGAACAC
P1-150-F	Forward	GACAAAGGCCGGCCAGAATTCCCAAACCTCGCCCTCATGC
P1-150-R	Reverse	GGCTCGAGAGGCCTTGAATTCTTATTGCTCCCCGCTATTAACATTAG
P1-140-R	Reverse	GGCTCGAGAGGCCTTGAATTCTTATAGTTGAGTGATCTGCACCTCACG
P1-130-R	Reverse	GGCTCGAGAGGCCTTGAATTCTTAAATTGACTGCTCCTGCTTTACTTG

### Epitope analysis

Structural modeling of the GAstV-1 TZ03 ORF2 P1 domain was conducted through the Phyre2 web-based platform for protein structure prediction. Antigenic determinants recognized by mAbs were mapped onto the predicted tertiary structure using PyMOL molecular visualization software. For comparative analysis, full-length ORF2 polypeptide sequences from representative astrovirus strains were obtained from the GenBank nucleotide repository. Multiple sequence alignments focusing on epitope-containing regions were executed with Lasergene MegAlign (DNASTAR bioinformatics suite) to evaluate the conservation and variation among different strains.

### Development of an indirect competitive ELISA (icELISA)

The purified ORF2 recombinant protein was coated onto the wells of a 96-well plate (Sangon, Shanghai, China) at various concentrations ranging from 0.125 to 2.0 μg/mL using carbonate-bicarbonate buffer, with three wells per concentration. The plate was incubated overnight at 4°C to allow adsorption. After adsorption, the plate was washed five times with 200 μl of PBST and blocked with 100 μl of 5 % skim milk in PBS (blocking buffer) at 37°C for 1 h. Following an additional five washes with 200 μl of PBST, the wells were incubated with a mixture of 50 μl GAstV-1 positive or negative serum (diluted 1:2 to 1:512 in blocking buffer) and 50 μl of mAb A5A1 (diluted 1:500 to 1:20,000 in blocking buffer) at 37°C for 2 h. Then, the plate was washed five times with 200 μl of PBST and incubated with 100 μl of HRP-labeled goat anti-mouse IgG (diluted 1:5,000 to 1:40,000 in blocking buffer) at 37°C for 1 h. After a final five washes with 200 μl of PBST, chromogenic development was initiated by the addition of 3,3′,5,5′-tetramethylbenzidine (TMB) enzymatic substrate. The enzymatic reaction was quenched through acidification with 50 μL 2 M H₂SO₄, followed by immediate spectrophotometric quantification at 450 nm wavelength. The reaction parameters were optimized based on the maximum negative-to-positive (N/P) ratio.

### Determination of the cut-off value for icELISA

The optimized icELISA procedure was employed to test 30 GAstV-1 negative serum samples. The percent inhibition (**PI**) values were calculated using the formula:

PI=(1−ODvalueofindividualsample/ODvalueofnegativecontrol)×100%

The mean PI values and standard deviations (**SD**) of the 30 negative serum samples were calculated. The PI cut-off value for positive samples in the icELISA was established as the mean PI value + 2 × SD (Zhang et al., 2022a).

### Evaluation of icELISA for GAstV-1

The sensitivity of the icELISA was evaluated using a series of dilutions of positive serum samples ranging from 1:4 to 1:512. The specificity of the icELISA was assessed using serum samples from various sources, including duck hepatitis A virus type 1 (**DHAV-1**), duck hepatitis A virus type 3 (**DHAV-3**), duck Tembusu virus (**DTMUV**), avian reovirus (**ARV**), H9N2 subtype avian influenza virus (**H9N2-AIV**), Newcastle disease virus (**NDV**), goose parvovirus (**GPV**), and GAstV-2. To evaluate the reproducibility of the icELISA, three positive goose serum samples and three negative goose serum samples were tested in triplicate.

### Comparison of icELISA with IFA

To evaluate the concordance between the icELISA and IFA, a total of 80 goose serum samples, sourced from different farms, were analyzed using both methods. The IFA procedure was performed as previously described, with the goose serum diluted tenfold serving as the primary antibody.

## Results

### Preparation of recombinant GAstV-1 ORF2 protein

**Fig. 1:**
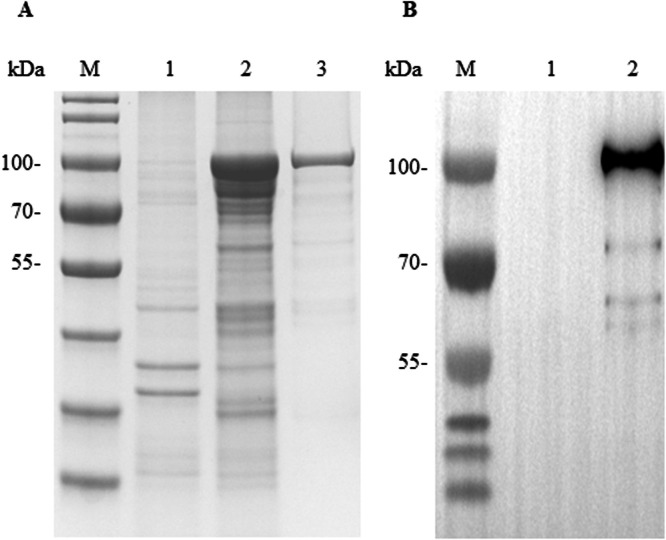
Expression and purification of goose astrovirus 1 (**GAstV-1**) ORF2 protein. (A) SDS-PAGE analysis of ORF2 recombinant protein expression. 1, Bacterial lysates from IPTG-induced *E. coli* BL21(DE3) containing pET-30a plasmid; 2, Bacterial lysates from IPTG-induced *E. coli* BL21(DE3) containing pET-ORF2 plasmid; 3, The purified recombinant ORF2 protein. (B) Western blotting analysis of purified recombinant ORF2 protein. The identity of the purified recombinant GAstV-1 ORF2 protein was verified by immunoblotting using a His-tag monoclonal antibody. 1, Bacterial lysates from IPTG-induced *E. coli* BL21 (DE3) transformed with pET-30a; 2, The purified recombinant ORF2 protein.

To produce the recombinant GAstV-1 ORF2 protein, the ORF2 gene was cloned into the pET-30a vector. Following IPTG induction, SDS-PAGE analysis confirmed the expression of a ∼100 kDa recombinant protein (Fig. 1A). Affinity purification utilizing nickel-charged magnetic beads yielded high-purity protein preparations (Fig. 1A). The concentration of purified recombinant ORF2 protein was 160 μg/mL. Immunoblotting assays with anti-His mAb exhibited distinct antigen-antibody interactions, confirming the integrity of the recombinant protein (Fig. 1B). These data collectively validate the successful production of immunoreactive GAstV-1 ORF2 protein for downstream applications.

### Generation and characterization of mAb specific to GAstV-1 ORF2 Protein

**Fig. 2:**
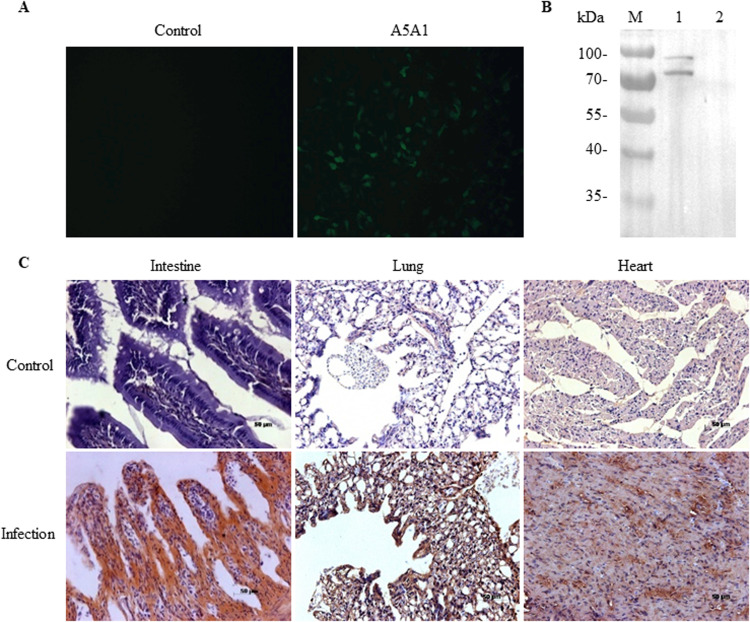
Characterization of the generated monoclonal antibody (**mAb**) A5A1 specific for goose astrovirus 1 (**GAstV-1**) ORF2 protein. (A) Immunofluorescence Assay (**IFA**) analysis. Goose embryonic kidney (**GEK**) cells were infected with the GAstV-1 TZ03 strain. Three days post-infection, cells were fixed and incubated with mAb A5A1 as the primary antibody. (B) Western blotting (**WB**) analysis. GEK cells were infected with the GAstV-1 TZ03 strain and harvested three days later. Cell lysates were subjected to WB analysis using mAb A5A1 as the primary antibody. 1, Lysate of GAstV-1-infected GEK cells; 2, Lysate of uninfected GEK cells. (C) Immunohistochemical analysis. One-day-old goslings were infected with the GAstV-1 TZ03 strain. After euthanasia, tissues from the small intestine, lung, and heart were collected and analyzed using mAb A5A1 as the primary antibody.

To generate mAbs against the GAstV-1 ORF2 structural protein, BALB/c mice were immunized with the purified ORF2 protein. Before cell fusion, the immunized mice demonstrated specific antibody titers of 1:64,000, significantly higher than those of the negative controls (data not shown). Following screening and subcloning, a hybridoma cell line, designated A5A1, was successfully established to stably produce mAbs targeting GAstV-1 ORF2. The A5A1 cell line was identified as producing IgG1 subclass antibodies with κ-type light chains. The specificity of the A5A1 was further characterized using various assays. IFA demonstrated that A5A1 specifically recognized GAstV-1 TZ03 strain-infected GEK cells (Fig. 2A). WB results revealed that two distinct specific bands, with approximate molecular weights of 75 kDa and 90 kDa, were exclusively detected in GAstV-1 TZ03 strain-infected GEK cells, whereas no such specific bands were observed in the control group cells (Fig. 2B). Additionally, IHC results showed that A5A1 selectively targeted GAstV-1 antigens in the small intestine, lung, and heart tissues of infected goslings (Fig. 2C), with no cross-reactivity observed in the control group. These results collectively confirm that the mAb A5A1 generated in this study exhibits strong and specific binding to the GAstV-1 ORF2 protein. This mAb thus holds great potential as a valuable tool for detecting GAstV-1 antigens in both *in vitro* and *in vivo* settings.

### Mapping of epitope recognized by mAb A5A1

**Fig. 3:**
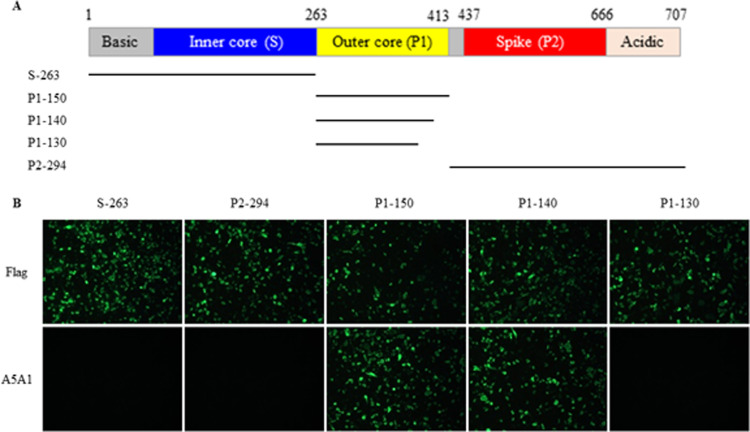
Mapping the B cell epitope in goose astrovirus 1 (**GAstV-1**) ORF2 protein recognized by the monoclonal antibody (**mAb**) A5A1. (A) Schematic of GAstV-1 TZ03 ORF2 protein domain structure. The ORF2 protein is composed of four structural domains: the S domain (blue), the P1 domain (yellow), the P2 domain (red), and the acidic domain (orange). (B) Immunofluorescence Assay (**IFA**) analysis of GAstV-1 ORF2 protein using mAb A5A1. 293 cells were transfected with recombinant plasmids containing various truncated fragments of the ORF2 protein. IFA was performed using mAb A5A1 or anti-FLAG mAb as the primary antibody to detect the expressed fragments.

To locate the B-cell epitope recognized by the mAb A5A1, various truncated fragments of the GAstV-1 ORF2 gene were cloned into recombinant plasmids and transfected into 293A cells (Fig. 3A). IFA was subsequently performed using A5A1 as the primary antibody. The results indicated that A5A1 specifically bound to 293A cells transfected with the P1 gene fragment, but not with the S or P2 gene fragments (Fig. 3B). This suggests that the epitope recognized by A5A1 is located within the P1 domain of the ORF2 protein. Further analysis revealed that A5A1 could recognize the P1-140 gene fragment but not the P1-130 gene fragment, indicating that the B-cell epitope recognized by A5A1 is precisely located at the sequence ^131^SNREVQITQL^140^ of P1 domain, which corresponds to ^394^SNREVQITQL^403^ of the full-length ORF2 structural protein.

### Analysis of epitope recognized by mAb A5A1

To elucidate the structural features of the B-cell epitope recognized by mAb A5A1, the 3D structure of the P1 domain of the GAstV-1 TZ03 ORF2 protein was predicted and analyzed using bioinformatics tools. As depicted in Fig.s 4A and 4B, the epitope ^131^SNREVQITQL^140^ recognized by A5A1 is situated on the surface of the P1 domain and adopts a β-sheet secondary structure.

**Fig. 4:**
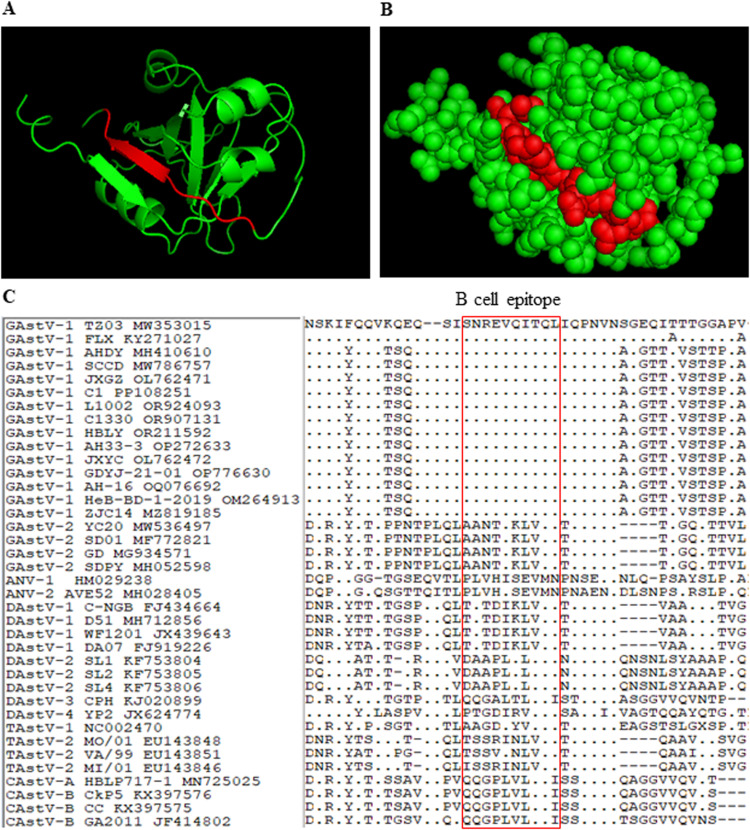
Analysis of the B cell epitope in goose astrovirus 1 (**GAstV-1**) ORF2 protein recognized by the monoclonal antibody (**mAb**) A5A1. (A, B) Three-dimensional structure of the P1 domain of GAstV-1 ORF2 protein. The 3D structure of the P1 domain was predicted using the Phyre online tool and visualized with PyMOL software. The cartoon (A) and sphere (B) representations highlight the epitope recognized by mAb A5A1 in red. The epitope ^131^SNREVQITQL^140^ is shown to be surface-exposed and adopts a β-sheet conformation. (C) Sequence alignment of the epitope recognized by A5A1. Sequence alignment of the epitope region (^131^SNREVQITQL^140^) from GAstV-1 with corresponding regions from various avian astrovirus strains, including GAstV-1, goose astrovirus 2 (**GAstV-2**), avian nephritis virus 1 (**ANV-1**), avian nephritis virus 2 (**ANV-2**), dusk astrovirus 1 (**DAstV-1**), dusk astrovirus 2 (**DAstV-2**), dusk astrovirus 3 (**DAstV-3**), dusk astrovirus 4 (**DAstV-4**), turkey astrovirus 1 (**TAstV-1**), turkey astrovirus 2 (**TAstV-2**), chicken astrovirus A (**CAstV-A**) and chicken astrovirus B (**CAstV-B**). The epitope region is highlighted in a red box.

To evaluate the homology of the B-cell epitope recognized by A5A1 across different avian astrovirus lineages, comprehensive sequence alignment was performed between the capsid protein of GAstV-1 strain TZ03 and corresponding viral components from various astrovirus species. The analysis demonstrated that the linear B-cell epitope ^131^SNREVQITQL^140^ identified by A5A1 exhibited complete sequence identity among all examined GAstV-1 isolates. However, comparative studies with other avian astrovirus subtypes, including GAstV-2, revealed substantial sequence variation in this antigenic region, with only 20-30 % amino acid similarity observed (Fig. 4C). This evidence indicates that the B-cell epitope recognized by A5A1 is highly conserved within GAstV-1 strains but is notably divergent from other astrovirus strains.

### Establishment of icELISA

The icELISA assay conditions were optimized according to the highest N/P value. The final experimental protocol involved coating the microplate with 100 μL of ORF2 protein solution (1 μg/mL) and incubating it at 4°C for 16 h. Subsequently, the plate was blocked with 100 μL of 5 % skim milk in PBS (blocking buffer) at 37°C for 30 min to eliminate non-specific binding sites. Serum samples were diluted 1:16 in the blocking buffer, while mAb A5A1 was diluted 1:2,000. The competitive reaction was carried out at 37°C for 2 h. HRP-conjugated goat anti-mouse IgG, diluted 1:10,000 in the blocking buffer, served as the secondary antibody. The reaction was developed with TMB at 37°C for 15 min and terminated with 50 μL of 2 N H₂SO₄.

The PI cut-off value for the icELISA was established using 30 negative serum samples. The average PI was 18.98 %, with a SD of 9.89 %. Based on these data, the PI cut-off value for icELISA was calculated as 38.76 % (Table 2). A sample with a PI value below 38.76 % was considered negative; otherwise, it was considered positive.

**Table 2:** Determination of the percent inhibition (**PI**) cut-off value of indirect competitive ELISA for goose astrovirus 1.

No.	PI					
1-6	29.58 %	27.90 %	24.08 %	22.30 %	26.08 %	27.82 %
7-12	30.21 %	22.68 %	25.66 %	17.99 %	23.68 %	6.91 %
13-18	9.93 %	10.33 %	7.76 %	13.57 %	19.65 %	6.69 %
819-24	34.17 %	6.56 %	9.76 %	15.24 %	10.06 %	31.65 %
25-30	6.63 %	37.54 %	35.76 %	9.04 %	9.99 %	10.08 %
Average PI	18.98 %					
standard deviations	9.89 %					
Cut-off value	38.76 %					

### Evaluation of icELISA

**Fig. 5:**
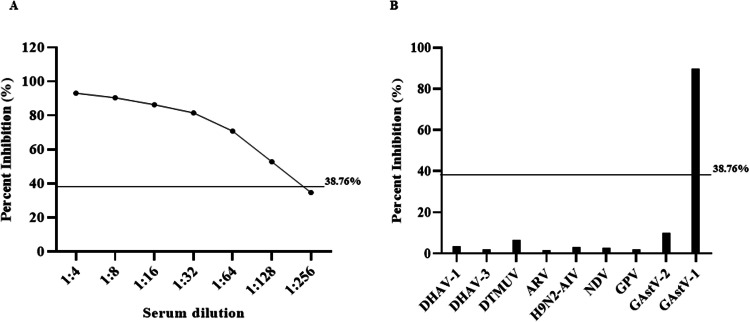
Sensitivity and specificity analysis of indirect competitive ELISA (**icELISA**) for goose astrovirus 1 (**GAstV-1**). (A) The sensitivity assessment of icELISA. Seropositive samples underwent serial dilution (1:4 to 1:256) for detection threshold determination. (B) Cross-reactivity assessment of icELISA. Specificity validation was conducted through parallel testing against duck hepatitis A virus type 1 (**DHAV-1**), duck hepatitis A virus type 3 (**DHAV-3**), duck Tembusu virus (**DTMUV**), avian reovirus (**ARV**), H9N2 subtype avian influenza virus (**H9N2-AIV**), Newcastle disease virus (**NDV**), goose parvovirus (**GPV**), goose astrovirus 2 (**GAstV-2**), and GAstV-1 antisera.

To evaluate the sensitivity of the icELISA, GAstV-1 positive serum was subjected to serial dilution and tested. The results (Fig. 5A) indicated that the PI value for the serum diluted at 1:128 was 52.81 %, while the PI value for the serum diluted at 1:256 was 34.75 %. This suggests that the limit of detection (LOD) for the icELISA is 1:128. To further validate the specificity of the icELISA, positive serum samples from various viruses, including DHAV-1, DHAV-3, DTMUV, ARV, H9N2-AIV, NDV, GPV, and GAstV-2, were tested. As depicted in Fig. 5B, all PI values were below the established cut-off, confirming that the icELISA does not cross-react with these sera and exhibits high specificity for GAstV-1.

To evaluate the robustness of the icELISA, six serum samples, including three positive and three negative, were chosen for intra-assay and inter-assay repeatability assessments. As indicated in Table 3, Table 4, the intra-assay coefficient of variation (**CV**) spanned from 1.74 % to 4.53 %, while the inter-assay CV ranged from 2.80 % to 9.95 %. These low CV values suggest that the icELISA exhibits excellent repeatability and reliability. Collectively, the findings highlight the superior sensitivity, specificity, and repeatability of the icELISA, confirming its potential as a reliable diagnostic tool for GAstV-1 detection.

**Table 3:** Intra-batch reproducibility test of indirect competitive ELISA for goose astrovirus 1.

Sample number	1^a^	2	3	Mean	SD^b^	CV %^c^
1#	90.95 %	92.53 %	95.69 %	93.05 %	0.0241	2.59
2#	87.21 %	86.54 %	81.26 %	85.00 %	0.0326	3.84
3#	74.60 %	72.25 %	75.50 %	74.11 %	0.0168	2.26
4#	3.22 %	3.10 %	3.14 %	3.15 %	0.0006	1.81
5#	12.19 %	11.17 %	11.92 %	11.76 %	0.0053	4.53
6#	5.03 %	4.96 %	5.13 %	5.04 %	0.0009	1.74

a percent inhibition value.

b Standard deviation.

c Coefficient of variation.

**Table 4:** Inter-batch reproducibility test of indirect competitive ELISA for goose astrovirus 1.

Sample number	1^a^	2	3	X	SD^b^	CV %^c^
1#	91.98 %	85.59 %	89.83 %	89.13 %	0.03253	3.65
2#	89.24 %	80.52 %	84.59 %	84.78 %	0.04364	5.15
3#	72.50 %	76.55 %	73.69 %	74.25 %	0.02081	2.80
4#	12.60 %	10.58 %	11.01 %	11.39 %	0.01063	9.33
5#	6.02 %	6.57 %	5.61 %	6.07 %	0.0048	7.92
6#	4.32 %	3.92 %	3.54 %	3.92 %	0.0039	9.95

a percent inhibition value.

b Standard deviation.

c Coefficient of variation.

### Comparison of icELISA with IFA

To evaluate the practicality of the icELISA, a total of 80 goose serum samples were tested using both icELISA and IFA. As shown in Table 5, icELISA yielded 35 positive and 45 negative results, whereas IFA detected 29 positive and 51 negative samples. When compared with IFA, icELISA demonstrated a positive concordance rate of 77.14 % (27/35) and a negative concordance rate of 95.56 % (43/45). Consequently, the overall concordance between icELISA and IFA was 87.5 % (70/80).

**Table 5:** Comparison of the indirect competitive ELISA (**icELISA**) and IFA using clinical goose serum samples.

		IFA		
		+	-	Total
icELISA	+	27	8	35
	-	2	43	45
	Total	29	51	80

## Discussion

The emergence of GAstV as a causative agent of gosling gout has posed significant challenges to the goose farming industry in China. While GAstV-2 continues to be the predominant strain in clinical outbreaks, recent epidemiological data indicate rising GAstV-1 prevalence, particularly during GAstV-2 co-infections (Chen et al., 2024; Zhou et al., 2024). Such co-infections exacerbate disease severity and increase mortality, highlighting the urgent need for GAstV-1-specific diagnostic tools to elucidate its disease contribution and enable targeted control.

Monoclonal antibodies are essential tools for both mechanistic studies and diagnostic development. Although five GAstV-2 ORF2-specific mAbs have been reported (Dai et al., 2022; Ren et al., 2022; Zhang et al., 2022b), no GAstV-1-specific mAbs had been developed prior to this study. Here, we generated the first GAstV-1 mAb (A5A1) using recombinant ORF2 protein expressed in *E. coli*. IFA and IHC confirmed A5A1’s specificity for GAstV-1-infected tissues, while WB analysis detected two specific protein bands at approximately 90 kDa and 75 kDa in infected cells. These molecular weights correspond to the immature and mature capsid proteins observed in HAstVs. Specifically, the mature protein VP70 (∼70 kDa) is generated through proteolytic cleavage of the immature precursor VP90 (∼90 kDa) by host proteases (Reuter et al., 2018; Ykema and Tao, 2021). Our findings suggest a conserved maturation mechanism for GAstV-1 structural proteins, highlighting the utility of A5A1 in detecting viral particles and their processed forms. However, the mAb A5A1 failed to neutralize GAstV-1 infectivity *in vitro* (data not shown), likely due to conformational differences between prokaryotically expressed ORF2 and native viral proteins. Similar limitations occurred in early HAstV mAb studies, where neutralizing epitopes required eukaryotic-expressed antigens or intact virions (Bogdanoff et al., 2017). Future efforts should prioritize eukaryotic expression systems (e.g., baculovirus or mammalian cells) to preserve native epitope conformations.

Monoclonal antibody-based epitope characterization provides critical insights for dissecting viral antigenicity and for designing targeted diagnostics and vaccines. In HAstVs, comprehensive epitope mapping studies have revealed that neutralizing antibodies predominantly target conformational epitopes within the hypervariable P2 domain of the ORF2 capsid protein, while linear epitopes are distributed across both the conserved S/P1 core and P2 spike regions (York et al., 2015; Espinosa et al., 2019). For avian astroviruses, however, epitope knowledge remains limited. While four B-cell epitopes have been identified in the S and P2 domains of GAstV-2 (Dai et al., 2022; Ren et al., 2022), no epitopes had been characterized for GAstV-1 prior to this work. To close this knowledge gap, we identified the first GAstV-1 epitope (^394^SNREVQITQL^403^) within the P1 domain of GAstV-1 ORF2. Structural modeling revealed that this epitope is surface-exposed, adopts a β-sheet conformation, and exhibits >95 % sequence conservation among GAstV-1 strains but marked divergence from other astroviruses, including GAstV-2. This significant divergence suggests that ^394^SNREVQITQL^403^ could serve as both a GAstV-1-specific diagnostic marker and subunit vaccine candidate. Furthermore, its linear nature (confirmed by reactivity with denatured proteins) enhances utility in WB and IHC assays compared to conformation-dependent epitopes requiring native protein folding.

Current diagnostic methods for GAstV-1 predominantly depend on nucleic acid-based techniques like RT-PCR and LAMP (Yu et al., 2020; Wang et al., 2022c). These methods are restricted to acute-phase detection and cannot assess herd immunity. To overcome this limitation, we developed an A5A1-based icELISA. The assay demonstrated high specificity (no cross-reactivity with GAstV-2, GPV, or other common avian viruses) and reproducibility (intra-/inter-assay CVs <10 %). Sensitivity analysis revealed a detection limit of 1:128 serum dilution, which is lower than the 1:6400 reported for virion-based iELISA (Zhang et al., 2023). This discrepancy likely stems from the differences in antigen presentation: whole virions present multiple copies of epitopes enhancing antibody avidity, whereas recombinant ORF2 lacks this multivalency. Despite this, our icELISA avoids the technical challenges and time-consuming processes associated with GAstV-1 propagation and purification. Unlike GAstV-2, which replicates efficiently in LMH cell lines, GAstV-1 lacks a robust *in vitro* propagation system, restricting cultivation to primary goose embryonic cells that yield typically low and unstable viral titers. This limitation has hampered establishment of a standardized virus neutralization test (**VNT**), making it unavailable as a reference standard for serological validation. While IFA provided useful serological correlates, field validation using 80 clinical sera revealed a GAstV-1 seropositivity rate of 43.75 %, consistent with prior epidemiological surveys (Zhang et al., 2023). Nevertheless, future work will focus on optimizing culture conditions and developing a VNT to definitively benchmark the diagnostic sensitivity and specificity of icELISA against a gold standard. This finding underscores the potential of icELISA as a valuable tool for herd-level surveillance of GAstV-1. To enhance its clinical utility, future improvements could focus on integrating GAstV-1 and GAstV-2 mAbs into a multiplex assay, thereby enabling the simultaneous detection of co-infections. Moreover, combining icELISA with broader serological surveys could elucidate GAstV-1’s zoonotic potential given astroviruses’ recognized cross-species transmission capacity (De Benedictis et al., 2011; Roach and Langlois, 2021; Huang et al., 2022).

In conclusion, this study provides the first GAstV-1-specific mAb (A5A1) and identifies a conserved linear epitope within the P1 domain of the ORF2 protein. The developed icELISA offers a reliable serological tool for GAstV-1 surveillance, addressing a significant gap in current diagnostic methods. These findings not only offer essential tools for further investigating the structure and function of the GAstV-1 ORF2 protein but also enhance our capacity to detect GAstV-1 antigens and antibodies.

## Funding

This investigation received financial support through the Science & Technology Innovation Team Program (grant number: NSF2023TC02) administered by Jiangsu Agri-Animal Husbandry Vocational College.

## CRediT authorship contribution statement

**Anping Wang:** Conceptualization, Project administration, Supervision, Writing – original draft, Writing – review & editing. **Li Liu:** Conceptualization, Supervision, Writing – original draft, Writing – review & editing. **Qingkang Zhou:** Methodology. **Xiaolu Zhang:** Validation. **Zhi Wu:** Formal analysis, Project administration, Software. **Shanyuan Zhu:** Data curation, Funding acquisition, Project administration, Resources, Supervision.

## Disclosures

The authors declare that they have no known competing financial interests or personal relationships that could have appeared to influence the work reported in this paper.
